# The Role of Probiotics in the Prevention of *Clostridioides difficile* Infection in Patients with Chronic Kidney Disease

**DOI:** 10.3390/nu16050671

**Published:** 2024-02-27

**Authors:** Sylwia Dudzicz-Gojowy, Andrzej Więcek, Marcin Adamczak

**Affiliations:** Department of Nephrology, Transplantation and Internal Medicine, Medical University of Silesia, 40-027 Katowice, Poland; sdudzicz@sum.edu.pl (S.D.-G.); awiecek@sum.edu.pl (A.W.)

**Keywords:** chronic kidney disease, *Clostridioides difficile* infection, probiotics

## Abstract

In patients suffering from chronic kidney disease (CKD), substantial unfavourable alterations in the intestinal microbiota composition, i.e., dysbiosis, have been noted. The main causes of such dysbiosis among others are insufficient dietary fibre content in the diet, fluid restrictions, medications used, and physical activity limitation. One clinically important consequence of dysbiosis in CKD patients is high risk of *Clostridioides difficile* infection (CDI). In observational studies, it was found that CDI is more frequent in CKD patients than in the general population. This appears to be related to high hospitalization rate and more often antibiotic therapy use, leading up to the occurrence of dysbiosis. Therefore, the use of probiotics in CKD patients may avert changes in the intestinal microbiota, which is the major risk factor of CDI. The aim of this review paper is to summarize the actual knowledge concerning the use of probiotics in CDI prevention in CKD patients in the context of CDI prevention in the general population.

## 1. Introduction

*Clostridioides difficile* infection (CDI) is one of the most frequent causes of post-antibiotic diarrhoea in hospitalised and quite recently also in non-hospitalized patients. Such infection is often characterized by severe clinical course and as a consequence leads to increased mortality. In the last decades, an increased frequency of occurrence and severity of CDI has been observed worldwide. Currently, CDI turns out to be the most common hospital-acquired infection affecting various groups of patients [1]. Chronic kidney disease (CKD) has grown into one of the most crucial global health problems. Related to that, CKD affects about 10% of the world’s population [2]. Intestinal dysbiosis and a high risk of developing CDI are often observed in CKD patients due to high co-morbidity, frequent infections, hospitalizations, antibiotic therapy, metabolic disorders, and dysbiosis. CDI occurs approximately twice as often in CKD patients than in the general population and is correlated with a higher incidence of severe forms of infection, complications, recurrence, and higher mortality [3]. Taking into account the above facts, CDI prevention in these patients seems to be extremely important. The main actions in CDI prevention are tightening hygiene procedures and reducing the impact of risk factors as much as possible. In recent years, in the general population, the possibility of intestinal physiological microbiota restoration using probiotics in the prevention of CDI has also been analysed, but the results of such clinical studies are inconclusive [4]. The intention of the following review paper is to present the available data on the prevention of CDI in patients with CKD using probiotics.

## 2. Methodology

**Figure 1:**
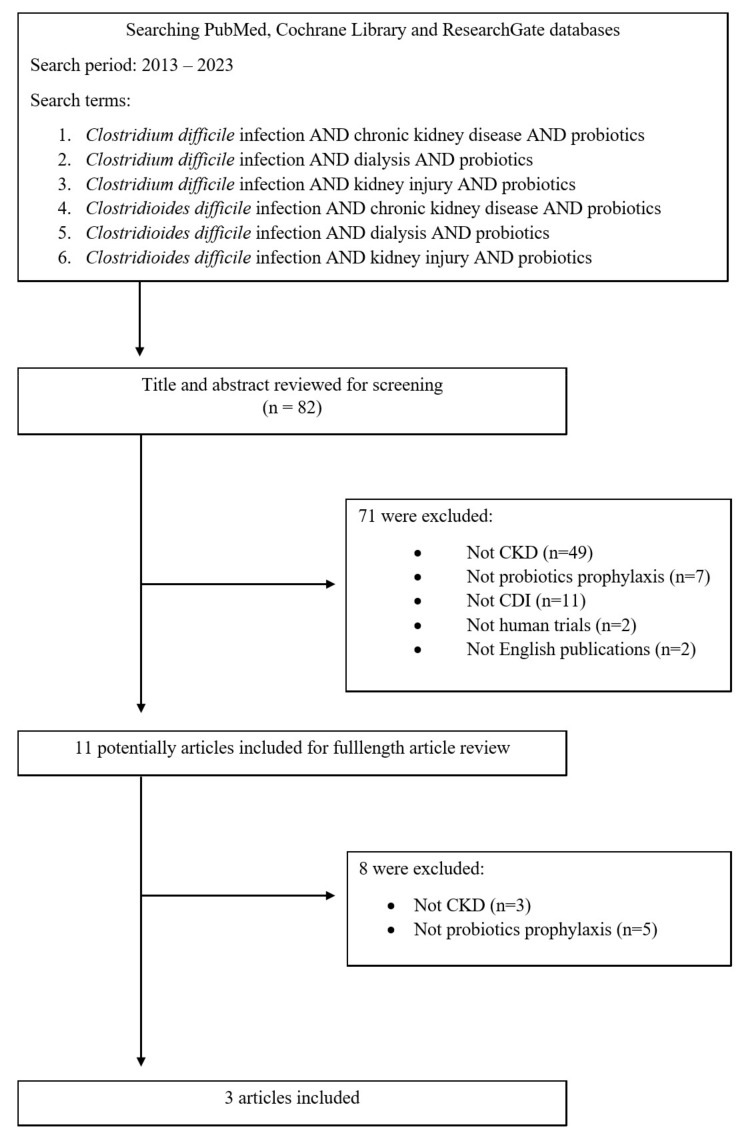
Flow chart of identification of eligible articles.

This literature review was based on the PubMed, Cochrane Library and ResearchGate databases using the keywords *Clostridium difficile* infection, *Clostridioides difficile* infection, chronic kidney disease, dialysis, kidney injury, probiotics. Original articles, review articles, and case reports from January 2013 to August 2023 were included in this analysis. Details are presented in the flow chart (Figure 1).

## 3. Dysbiosis in Chronic Kidney Disease Patients

During chronic kidney disease (CKD) progression, the plasma concentration of uremic toxins, including urea, increases. It was shown that high urea plasma concentration increases gut permeability, enabling the movement of pathogenic microorganisms from the intestinal lumen into the bloodstream, leading to chronic, subclinical inflammation [5]. The consequences of such inflammation are vascular endothelium damage and accelerated atherosclerosis, clinically manifested by cardiovascular disease, which is one of the major reasons of premature death among CKD patients [6]. In addition, the high urea and uric acid serum concentrations also increase their concentrations within the intestinal lumen. This promotes the multiplication of bacterial species, produces uricase, urease, and enzymes, and metabolizes indole and phenolic compounds in order to reduce the concentration of urea by converting it to ammonia [7]. However, increased production of ammonia in the intestinal lumen leads to a pH increase, facilitating the growth of pathogenic bacteria and dysbiosis [8]. In CKD patients, an increased *Firmicutes*, *Actinobacteria*, and *Proteobacteria* count and a decreased *Bifidobacteria* and *Lactobacillus* count were found [9]. Urea is also converted into ammonium hydroxide, which degrades tight junction proteins. The results of an experimental study in rats after subtotal nephrectomy as well as rats with adenine-induced chronic tubulointerstitial nephritis showed a significant reduction in expression of claudin and occluding epithelial tight junction molecules in the intestine [10,11]. Insufficient intake of plant fibre and a low-potassium and low-phosphate diet, recommended often to CKD patients, also may reduce *Bifidobacteria* population in the intestine and consequently decrease short-chain fatty acids production by microbiota [12]. Short-chain fatty acids are, among others, the source of energy for intestinal epithelial cells. A reduction in the bioavailability of short-chain fatty acids in intestinal epithelial cells may cause abnormalities in the intestinal barrier [13].

In CKD patients, frequent occurrence of constipation and defective gastrointestinal motility have been observed due to insufficient dietary fibre content in the diet, fluid restrictions, certain medications, and limitations in physical activity. Slowing down the intestinal passage can lead to unfavourable bacterial growth in the intestinal lumen [14].

One of the possibilities to counteract the occurrence of dysbiosis and obtain beneficial modifications of the intestinal microbiota composition is the use of probiotics. In interventional studies in CKD patients, a decreased serum concentration of pro-inflammatory cytokines and uremic toxins, improvements in digestive system function (decreased number of episodes of anorexia, nausea, vomiting, heartburn, stomach ache, bloating, constipation, and diarrhoea), and improvement in the quality of life were found during administration of probiotic preparations [15].

Probiotics are live microorganisms which provide health benefits to the host organism. The most important condition that probiotic preparations must fulfil is using a non-pathogenic strain that does not contain genes for resistance to the antibacterial drugs. The origin of the strain must be well documented, along with its mechanism of action and the safety of its use confirmed in interventional clinical studies. To perform their basic functions, probiotic strains of bacteria must have mechanisms of resistance to digestive enzymes and low pH, the ability to colonise the intestinal mucosa and growth in the conditions prevailing in the human intestine, and be able prevent the colonisation of the intestine by pathogenic microorganisms [16]. Probiotic preparations contain one or many strains of bacteria. The pharmaceutical form of probiotics must allow the survival of bacteria during intestinal transit. Probiotics most often are in the form of lyophilised capsules or tablets that are resistant to gastric acid, bile, and digestive enzymes. The preparation should contain 10^9^–10^10^ colony-forming units (CFUs) of live bacteria in a single dose. Classic probiotic microorganisms contain representatives from genera such as *Lactobacillus*, *Bifidobacterium*, *Streptococcus*, and fungi genus *Saccharomyces* [17].

## 4. *Clostridioides difficile*

*Clostridioides difficile* infection (CDI) is one of the major causes of post-antibiotic diarrhoea in hospitalised and recently also non-hospitalized patients [18]. *Clostridioides difficile* is spread via the faecal–oral route with spores. Due to the fact that *Clostridioides difficile* is an obligate anaerobic bacillus; therefore, in order to be able to transfer between organisms it had to develop the ability to produce spores resistant to oxygen. Spores are also able to survive in contact with high temperatures, acids, most disinfectants, and antibacterial drugs. The spore is composed of a dehydrated core containing DNA, tRNA, ribosomes, and enzymes surrounded by several layers of membranes with various physicochemical properties that determine their resistance [19]. In the duodenum, spore forms exposed to the conjugated bile acids are transformed into vegetative forms, which start multiplying in the intestinal lumen [20]. *Clostridioides difficile* pathogenicity is determined by the possibility of producing toxins A and B and binary toxins from the vegetative form of this bacteria. These toxins inactivate Rho guanosine-5’triphosphatase (GTPase) and, as a consequence, lead to depolymerisation of actin fibres and colonic epithelial cell damage. As a result, an excessive production of mucus, diarrhoea, colitis, and the formation of the so-called pseudomembranes consisting of leukocytes, bacteria, fibrin, and necrotic epithelial cells frequently occurs [21,22].

Typical clinical symptoms of CDI are watery diarrhoea, pain in the lower abdomen, fever, malaise, nausea, and vomiting [23]. The clinical picture of CDI infection in some patients is characterized by colitis without the formation of pseudomembranes, pseudomembranous colitis, fulminant colitis complicated by acute *megacolon toxicum*, paralytic intestinal obstruction, or colon perforation [24]. The necessary conditions for the diagnosis of CDI are the presence of diarrhoea or megacolon toxicum with identification of toxins A and/or B in the stool or demonstration of the presence of a toxigenic strain of *Clostridioides difficile* in stool culture or detection of pseudomembranous colitis during endoscopic examination, surgery, or histopathological examination [25]. CDI recurrence can be diagnosed when the infection recurs within eight weeks of the previous episode. Approximately 20% of CDI patients experience a relapse after treatment, and mortality increases in subsequent relapses. CDI recurrences may occur due to unstable colonization of the intestines with physiological bacterial flora, persistent *Clostridioides difficile* spores in enterocytes, insufficient production of IgG antibodies against toxins A and B, and *Clostridioides difficile* resistance to antibiotics [26]. A stool sample or, in the absence of diarrhoea, a rectal swab is taken from a patient suspected of having CDI. The sample is sent to the laboratory within 2 h of collection, and if this is not possible, it should be stored at 4 °C for no longer than 72 h. In CDI diagnosis, various methods are used: enzyme-linked immunosorbent tests detecting toxins A and B, nucleic acid amplification tests (NAAT), toxigenic culture (TC), enzyme-linked immunosorbent assay glutamate dehydrogenase (EIA GHD), and cell cytotoxicity neutralization assay (CCNA). Currently, a multi-stage diagnostic algorithm is recommended for the diagnosis of CDI. At the first stage, high-sensitivity tests are recommended, such as NAAT or EIA GDH. If the result is positive, in the second stage, enzyme-linked immunosorbent tests are performed to detect toxins A and B. If these tests are positive, the presence of CDI may be confirmed. If there is a negative result of this test in the presence of clinical symptoms of CDI, it should be considered to complete an NAAT test (in cases where EIA GDH was performed in the first step) or stool culture for *Clostridioides difficile* with determination of its toxicity to fully confirm or exclude the diagnosis of CDI [25].

The following components contribute to CDI prevention: intensification of hygiene activities, elimination of risk factors, and therapeutic interventions modifying the intestinal microbiota. To increase the general level of hygiene, it is recommended to often wash hands with soap and water, take a shower frequently, lower the seat before flushing the toilet, and isolate a patient with CDI during the period when diarrhoea occurs and for 48 h after it subsides. These actions are intended to reduce the spread of spores. Medical staff should use disposable gloves and aprons when initiating contact with a CDI patient. Appropriate disinfection of surfaces (agents containing hypochlorite at a concentration of ≥1000 ppm), medical equipment, and objects used by the patient, including bedding, should also be undertaken [27]. Another important element in preventing CDI is reducing the impact of risk factors. It is recommended to use antibiotics only when clearly indicated, as the increased risk of CDI persists for up to 3 months after the end of antibiotic therapy. If antibiotic therapy is necessary, it is recommended to choose an antibiotic with a lower CDI risk, such as carbapenems, macrolides, sulphonamides, aminoglycosides, tigecycline, rifampicin, metronidazole, and vancomycin. The use of non-steroidal anti-inflammatory drugs, proton pump inhibitors, or histamine 2 receptor antagonists should be limited only to clinical situations in which there are absolute indications to start treatment with these preparations and only for a strictly defined period of time [28]. The easiest method of therapeutic intervention intended to modify intestinal microbiota composition is probiotics use. The mechanisms of CDI prevention with probiotics include adhesion to intestinal mucosa cells, stimulated mucins production by intestinal mucosa cells, production of antibacterial substances, inhibition of the development of vegetative forms of *Clostridioides difficile* by deconjugating bile acids, local stimulation of the immune system activity, and competition for nutrients and places of colonization with pathogenic organisms [29]. The use of probiotics in the prevention of CDI is discussed in more detail in the next paragraphs of this article.

Current treatment guidelines for CDI were published in 2017 by *the Infectious Diseases Society of America* (IDSA) and the *Society of Healthcare Epidemiology of America* (SHEA). Depending on the severity of the infection and the occurrence of CDI recurrence, there are several types of recommended algorithms. The main drugs used to treat CDI are vancomycin and fidaxomicin administered orally. For patients with CKD, the same treatment as for the general population is recommended. It is also worth noting that vancomycin and fidaxomicin are not absorbed in the gastrointestinal tract; therefore, there is no need to modify the dose of the drug in a patient with renal insufficiency [30].

### 4.1. Clostridioides difficile Infection in CKD Patients

As previously discussed, CKD patients had higher CDI risk than the general population. It has also been documented that CDI in CKD patients, especially those treated with renal replacement therapy, is more severe, increases complications and mortality risk, prolongs hospitalisation, and increases treatment costs [31]. Phatharacharukul et al., in a meta-analysis including 20 clinical trials (case–control, cohort, and cross-sectional studies) involving 162,218,041 patients, demonstrated significantly increased CDI risk in patients with CKD and end-stage renal disease (ESRD) than in the general population—RR 1.95 (95% CI; 1.81–2.10) and RR 2.63 (95% CI 2.04–3.38), respectively. Moreover, they also found a higher incidence of CDI relapses in CKD patients (RR 2.61, 95% CI 1.53–4.44) [32]. A meta-analysis by Thongprayoon et al. (including case–control studies and 18 cohort studies involving 116,875 patients) found a higher risk of severe forms of CDI or complicated CDI in CKD patients (RR 1.51, 95% CI; 1.00–2.28). Additionally, the incidence of infection relapses and death due to CDI was higher in these patients (2.73-fold and 1.76-fold increased risks, respectively) [33]. In different meta-analyses, including four cohort studies with 8,214,676 patients, they found significantly increased risks of mortality in CKD and ESRD patients during CDI (RR 1.73, 95% CI 1.39–2.15 and RR 2.15, 95% CI; 2.07–2.23, respectively) [34]. In single-centre, retrospective case–control study including 513 patients, Kim et al. found that patients in stages CKD 4 and CKD 5 or dialysis ESRD patients had an increased CDI risk (OR = 2.90 and OR = 3.34, respectively) [35]. Abdelfatah et al. showed in a multivariate analysis that most CDI relapses are diagnosed in patients with comorbidities, including CKD (OR1.3; 95% CI, 1.0–2.4; *p* = 0.039) [36].

A higher CDI risk in CKD patients is due to more frequent hospitalisation, more frequent infections, and the resulting more frequent antibiotic therapy, usually multidrug antibacterial pharmacotherapy, leading to the occurrence of dysbiosis, as well as malnutrition with hypoalbuminemia and immune system disorders associated with uraemia [37].

Therefore, it seems the use of probiotics might avoid changes in the intestinal microbiota (i.e., dysbiosis), which may lead to the occurrence of CDI in CKD patients.

### 4.2. Use of Probiotics in the Prophylaxis of Clostridioides difficile Infections in Chronic Kidney Disease Patients

During the literature review, out of 82 initially selected articles, only 3 discussed CDI prevention using probiotics in CKD patients. Detailed exclusions of articles during the literature review process are presented in Figure 1. Details of the search criteria are already described in the methodology section of the paper.

Three review articles (two of them were published by Dudzicz et al. from our group) concerning CDI prophylaxis with *Lactobacillus plantarum 299v* (LP299v) in hospitalised patients in the nephrology and transplantation ward were published [38,39,40]. The results discussed in these review articles were related to a retrospective analysis of the cohort consisting of 5341 hospitalized patients during three consecutive one-year periods published by Dudzicz et al. [41]. A reduced incidence of CDI was found during the period with the use of a probiotic preparation containing LP299v in a group of high-risk patients (patients during simultaneous antibiotics therapy and immunosuppressive therapy). In the study group, CKD patients constituted 62% of the population and chronic dialysis patients 9%. During the first observation period, patients received probiotics containing various bacterial strains: *Bifidobacterium lactis*, *Lactobacillus acidophilus*, *Lactobacillus delbrueckii*, *Lactobacillus rhamnosus*, and *Saccharomyces boulardii*. In the second period, all subjects from the risk group received LP299v, and then in the third period of observation, this method of prophylaxis was ended and the previous strains were resumed. As presented in Table 1, a significant decrease in CDI incidence was observed during prophylaxis with LP299v, followed by a significant increase in incidence after cessation of this method of prophylaxis (RR 0.11; CI 0.03–0.47; *p* = 0.0003 and RR 6.93; CI 1.58–30.47; *p* = 0.0028). During prophylaxis with LP299v, no severe CDI clinical symptoms were observed. During LP299v prophylaxis, two relapses of CDI were diagnosed, but the severity of the gastrointestinal symptoms during these was mild. The economic aspect of effective CDI prevention also was analysed in this study. It was calculated that to prevent a CDI case in one hospitalized patient, the LP299v strain need be administered to 15 patients (i.e., number needed to treat is 15). The cost of such prophylaxis method was 17.5 PLN (4.1EUR) per patient for the entire duration of prevention, on average 14.7 days. In consequence, the cost of prevention for one case of CDI is 262.5 PLN (61.5EUR) [42]. For comparison, the costs of treating one case of CDI ranges from USD 8911 to USD 30,049 for one hospitalized patient [43].

## 5. *Lactobacillus plantarum 299v*

Due to demonstrated high effectiveness of LP299v in CDI prevention in CKD patients, a brief description of this strain is presented below. LP299v are Gram-positive lactic acid bacteria that naturally occur on the mucosa of the human gastrointestinal tract. LP299v has a high ability to colonize the intestinal mucosa related to the specific mannose-binding mechanism of adhesion of this strain to intestinal epithelial cells [44]. Moreover, these properties also prevent the adhesion of potentially pathogenic organisms to the intestinal epithelium. LP299v adheres to intestinal epithelial cells by binding to mannose residues and increases the production of mucin, which inhibits the adhesion of pathogenic microorganisms [45].

The safety of use of the species *Lactobacillus plantarum* is confirmed by its inclusion on the Qualified Presumption of Safety List of the European Union. In order for a microorganism to be included on the above-mentioned list, it must meet many restrictive conditions, including having a well-defined taxonomy, safety of use confirmed in research, lack of pathogenic properties (e.g., the possibility of producing toxins), and an acceptable antibiotic resistance profile [46]. The use of this strain was studied in patients at high risk of infections, e.g., patients in intensive care units, paediatric population from 12 months of age, including populations after bone marrow transplantation, and patients after major abdominal surgeries, and no significant side effects were observed [42,47,48,49].

The clinical advantage of the LP299v strain is the alleviation of gastrointestinal symptoms such as nausea, pain, or diarrhoea intensity during diarrhoea associated with antibiotic therapy or CDI [50]. In patients with irritable bowel syndrome, a significant improvement in symptoms was observed during administration of LP299v: with a significant reduction in abdominal pain (0.68 + 0.53 vs. 0.92 + 0.57, *p* < 0.05), a significant reduction in the frequency of bowel movements (1.01 + 0.77 vs. 1.71 + 0.93, *p* < 0.05), and a reduction in bloating [51].

LP299v also has immunomodulatory properties, among others, reducing the serum concentration of pro-inflammatory cytokines IL-6, IL-8 and IL-12 [52,53]. LP299v appears to modulate the immune system depending on the possibility of infection, but it may also decrease the immune response in patients with persistent inflammation [54]. Another interesting property of the LP299v strain is its ability to increase the iron absorption from the gastrointestinal tract. Bering et al. assessed the possibility for increased non-haem iron absorption after administration of LP299v. The iron absorption from four different oat gruels was assessed. In the case of oat gruel combined with LP299v, a significant increase in iron absorption of 80% was found compared to the others tested (*p* < 0.0001) [55].

## 6. Probiotics in the Prevention of *Clostridioides difficile* Infection in General Population

**Figure 2:**
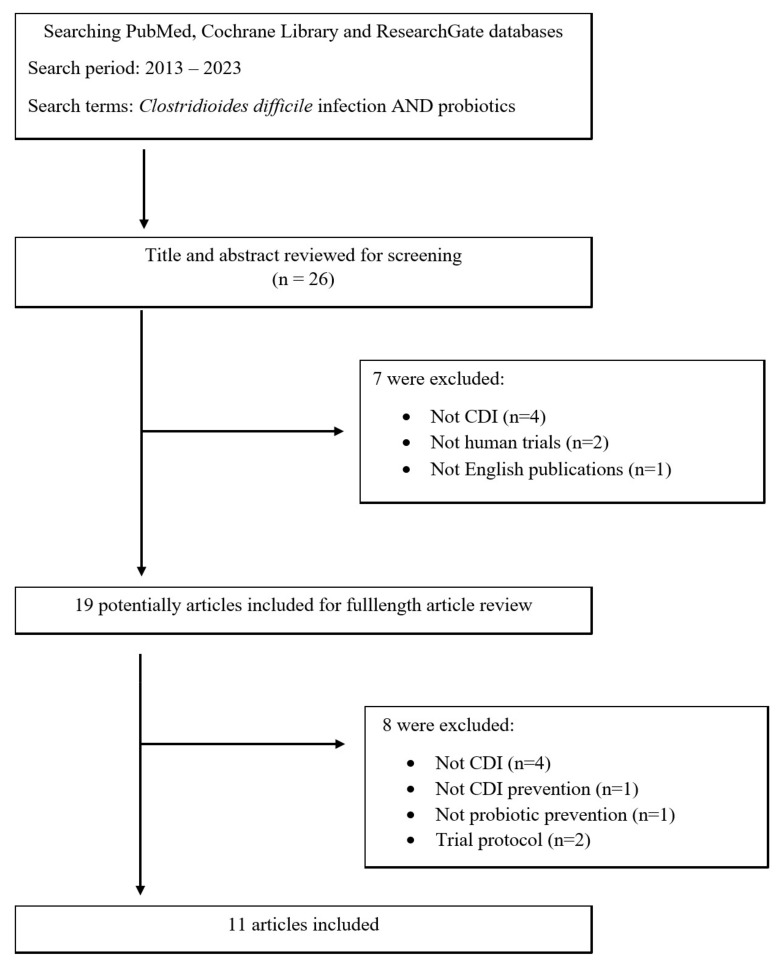
Flow chart of identification of eligible articles.

Due to the limited amount of data on the use of probiotics in the prevention of CDI in the population of patients with chronic kidney disease, it was decided to analyse the available data on the general population. The review was based on the PubMed, Cochrane Library, and ResearchGate databases using the keywords *Clostridioides difficile* infection and probiotics. Only original articles on clinical and randomized controlled trials from January 2013 to August 2023 were included in this analysis. The details are presented in the flow chart (Figure 2).

In the analysed articles, the most frequently mentioned group of probiotic bacteria is the *Lactobacillus* species. This species is also part of the human microbiome and is one of the largest populations of bacteria found in the human body [56]. *Lactobacillus* spp. bacteria are one of the groups with the best-studied probiotic properties. *Lactobacillus* have been used in fermented dairy products for many years. They are widely used in intestinal disorders in both the elderly and children populations [57]. *Lactobacillus* spp. are classified by the Agriculture Organization of the United Nations and World Health Organization as “generally regarded as safe” organisms [58]. In rare cases, they can cause infection in humans, which manifests as bacteraemia or endocarditis, but this mainly happens in immunocompromised patients [57]. In the selected articles, the following representatives of *Lactobacillus* spp. were used as CDI prophylaxis: *Lactobacillus acidophilus*, *Lactobacillus rhamnosus GG*, *Lactobacillus casei*, and *Lactobacillus reuteri*.

The PLACIDE study, one of the largest (2941 patients) randomised clinical trials analysing probiotics use for CDI prevention, did not observe that use of probiotic mixtures consisting of four strains of bacteria: *Lactobacillus acidophilus CUL60*, *Lactobacillus acidophilus CUL21*, *Bifidobacterium bifidum CUL20*, and *Bifidobacterium lactis CUL34*, decreases the risk of CDI [59]. Moreover, Box et al. evaluated the effectiveness of probiotics on CDI rates in 1576 hospitalised patients receiving antibiotics and found no significant differences between patients who received probiotics and those who did not (1.8% vs. 0.9%; *p* = 0.16). In this study, a probiotic mixture containing *Lactobacillus acidophilus CL1285*, *Lactobacillus casei LBC80R*, and *Lactobacillus rhamnosus CLR2* was used [60]. Dionne et al. in their prospective nested cohort study analysed the effect of using *Lactobacillus rhamnosus GG* as ventilator-associated pneumonia prophylaxis compared to placebo. One of the secondary outcomes the authors obtained was the incidence of CDI in these two groups. A total of 2650 mechanically ventilated patients participated in the study. In this study, there was no difference in the incidence of CDI in the ICU between the studied groups (interventional group 2.4% vs. control group 2.1%; *p* = 0.60) [61]. Rauseo et al. in a prospective, double-blinded, randomized placebo-controlled trial analysed the effectiveness of *Lactobacillus rhamnosus GG* use among patients receiving broad-spectrum antibiotics. In total, 88 patients were enrolled in this study; 44 patients in the study group received 1 capsule containing 1 × 10^10^ cells of *Lactobacillus rhamnosus GG* twice daily and 44 patients received placebo in the control group. The aim of the study was to analyse changes in the intestinal microbiota and colonization by antimicrobial-resistant organisms after exposure to antimicrobial agents, including *Clostridioides difficile*. There was no reduction in *Clostridioides difficile* colonization in patients after *Lactobacillus rhamnosus* was used in the interventional group (27% in interventional group vs. 14% in control group; OR 0.42 (95% CI 0.14–1.25); *p* = 0.11 at enrolment and after enrolment) [62]. Rajkumar et al. in a multicentre, double-blind, placebo-controlled, randomized trial assessed the effectiveness of a preparation containing *Lactobacillus casei DN114001* in reducing the incidence of antibiotic-associated diarrhoea (AAD) and CDI. The study included 1127 patients on antibiotic therapy aged over 55 years randomized to the probiotic group or placebo group. Participants took an oral probiotic as a yoghurt drink or placebo twice daily for up to a week after completing antibiotic therapy. AAD was observed in 19.3% of the probiotic group vs. 17.9% of the placebo group (OR 1.10, 95% CI 0.82–1.49, *p* = 0.53). CDI was found in 8 cases of the probiotics group and 11 of the placebo group. Taking into account these results, no significant evidence of the beneficial effect of this method in prophylaxis was found [63]. The effectiveness of the *Lactobacillus casei* strain in the prevention of AAD and CDI was also analysed by Alberda et al. [64]. In total, 32 patients hospitalized in the Intensive Care Unit were included in the study. They received a probiotic drink containing 1 × 10^9^ CFU of *Lactobacillus casei DN-114 001* twice daily orally or by feeding tube. AAD was documented in 12.5% of the probiotic group and 31.3% in the control group (*p* = 0.394). CDI was diagnosed in one patient in the probiotic group compared to three in the control group (*p* = 0.6) [64]. Kołodziej et al. assessed the effectiveness of the *Lactobacillus reuteri* DSM 17938 strain in the prevention of antibiotic-associated diarrhoea (AAD) in the paediatric population. In total, 250 children receiving antibiotic therapy were randomized to the study or control group. Both groups received an appropriate preparation containing *Lactobacillus reuteri* or a placebo twice daily for up to a week after completing antibiotic therapy. The AAD occurrence was 11.4% in the probiotic group compared with 6.5% in the placebo group (ARR −0.05 (−0.13 to 0.02)). The CDI occurrence was 0% in the probiotic group compared with 0.8% in the placebo group (ARR 0.01 (−0.02 to 0.04) [65].

Sadahiro et al. compared the effectiveness of oral antibiotics and probiotics in preventing infection after elective colon cancer surgery. In total, 310 patients with colon cancer were randomized to one of three groups: receiving probiotic prophylaxis, oral antibiotic prophylaxis, and without any infection prophylaxis. The probiotic preparation in this study was a *Bifidobacterium bifidum* strain. During the study, stool samples were collected 9 and 2 days before and 7 and 14 days after the surgery to determine the amount of bacteria and *Clostridioides difficile* toxins. The detection rates of the CD toxin before the operation were 2.0%, 5.1%, and 2.1% in groups A, B, and C, respectively (*p* = 0.48) and 14 days after surgery were 7.0%, 9.1%, and 10.5% in groups A, B, and C, respectively (*p* = 0.69). It is important to note that none of the patients from groups A, B, and C developed CDI. Additionally, in the postoperative periods, *Clostridioides difficile* proliferation seemed to be inhibited in Group A [66].

The single-celled yeast *Saccharomyces boulardii* is also used in CDI prevention. It is a probiotic with wide use in gastrointestinal disorders. The advantages of this probiotic in the prevention of CDI are low sensitivity to variable pH, which allows *Saccharomyces boulardii* to reach the large intestine after oral administration in an unchanged form, production of serine protease, which inhibits toxins A and B produced by *Clostridioides difficile*, and resistance to antibacterial agents causing CDI, so they do not affect the colonization of the intestine by *Saccharomyces boulardii* [67]. Carstensen et al. in a one-year controlled prospective intervention study assessed *Saccharomyces boulardii* effectiveness in CDI prophylaxis. The study included 1389 patients taking antibiotics from four hospitals in Denmark. The monthly incidence CDI rate decreased from 3.6% in the period before the implementation of *Saccharomyces boulardii* to 1.5% during the use of this method of prophylaxis. Additionally, the use of *Saccharomyces boulardii* prophylaxis was associated with a reduced CDI risk in all hospitals (OR = 0.06, 95% CI 0.02–0.16). In the two hospitals constituting the control group, the incidence rates for CDI did not change [68].

Prophylactic use of probiotics theoretically is a hopeful idea for CDI prophylaxis but the results of meta-analysis clinical studies are ambiguous. A meta-analysis by Goldenberg et al., including 31 randomised controlled trials with 8672 patients, found that the simultaneous use of probiotics during antibiotic therapy reduces the CDI risk by 60% [69]. In a systematic review with meta-regression analysis of 19 randomised controlled trials, comprising 6261 patients, Shen et al. found that supplying probiotic preparations near to the first antibiotic dose decreased the CDI risk by over 50% in hospitalised patients [70]. In another meta-analysis of 20 trials including 3818 participants, probiotics reduced CDI incidence by 66% [71]. The following probiotic strains were used in the analysed studies: *Lactobacillus acidophilus*, *Lactobacillus casei*, *Lactobacillus rhamnosus*, *Lactobacillus bulgaricus*, *Saccharomyces boulardii*, *Bifidobacterium lactis*, and *Streptococcus thermophiles*. The above-mentioned meta-analyses of interventional studies and observational studies did not specify the group of patients with CKD.

Another option used in the prevention of CDI, especially its recurrent forms, are live biotherapeutic products. As defined by the Food and Drug Administration are live biotherapeutic products that contain live organisms, such as bacteria, and are applicable to the prevention, treatment, or cure of a disease or condition of human beings. They are not vaccines [72]. The current analysis included two studies assessing the effectiveness of live biotherapeutic products in CDI prevention. In the randomized, double-blind, placebo-controlled, phase 3 study PUNCH CD3, 267 patients who had one or more CDI recurrences with a positive stool assay and who were previously treated with standard-of-care antibiotics participated. They were randomly assigned 2:1 to receive a single-dose enema of RBX2660 or placebo. BX2660 is a live biotherapeutic product consisting of a broad consortium of microbes prepared from human stool. The treatment success rate was 70.6% with RBX2660 versus 57.5% with placebo. The incidence of treatment adverse events was higher in the RBX2660 group compared with placebo and was mostly a higher incidence of mild gastrointestinal events [73]. In another double-blind placebo-controlled phase 2 clinical trial, the efficacy of two different doses of VE303, a defined bacterial consortium of eight commensal *Clostridia* strains, and placebo, in preventing the recurrence of CDI among adults at high risk of recurrences was analysed; 78 patients participated in the study. The CDI recurrence rates after eight weeks were 13.8% for high-dose VE303, 37.0% for low-dose VE303, and 45.5% for placebo, respectively (*p* = 0.006, high-dose VE303 vs. placebo; *p* = 0.3 low-dose VE303 vs. placebo) [74].

Currently, different guidelines are divergent regarding the use of probiotics for the primary prevention of CDI. *European Society of Clinical Microbiology and Infectious Diseases* (ESCMID) guidelines state that standard application of probiotics to avoid CDI during antibiotic therapy is not universally recommended. Recommendations concerning probiotics use are strongly recommended but have a low quality of evidence [75]. The *Infectious Diseases Society of America* (IDSA) does not introduce recommendations regarding probiotics. In this document, it is stated that there are insufficient data at this time to recommend the administration of probiotics for primary prevention of CDI outside of clinical trials [30]. The *American College of Gastroenterology* (ACG) recommend against probiotics for the prevention of CDI in patients being treated with antibiotics as a primary prevention method [76].

## 7. Summary

To sum up, the risk of CDI infection is high in CKD patients. Therefore, CDI prevention is important in these patients from both a clinical and epidemiological point of view. The results of several studies concerning the use of probiotics for CDI prevention in the general population are inconclusive. In addition, despite attempts to find a probiotic strain with high effectiveness in CDI prevention, currently the results of these studies do not allow us to establish clear guidelines for probiotic therapy in CDI prophylaxis. The main probiotic organisms showing best features in this direction are bacteria from the *Lactobacillus*, *Bifidobacterium*, and *Streptococcus* groups and *Saccharomyces boulardii* yeasts. Further studies are needed to select a specific strain with the best effectiveness in preventing CDI. A promising and future direction is live biotherapeutic products, which seem to be an interesting and effective alternative to faecal microbiota transplant in the prevention of recurrent forms of CDI. Moreover, as the literature review showed, there are only very few studies or review articles on CDI prevention among CKD patients. Future clinical studies analysing of the effectiveness of such prevention and aiming to select the most effective probiotic strains could bring significant medical benefits for CKD patients and economic benefits for hospital units and are undoubtedly needed.

## Figures and Tables

**Table 1:** The incidence of CDI before, during, and after cessation *Lactobacillus plantarum 299v* prophylaxis (based on the results of Dudzicz et al.) [41].

	(*n*)	(% All Hospitalized Patients)	vs. Incidence during Prophylaxis of LP299v
**Before introduction of LP299v**	18	1.03%	*p* = 0.0003
**During prophylaxis of LP299v**	2	0.11%	-
**After cessation of LP299v**	14	0.77%	*p* = 0.0028
